# Lysyl Hydroxylase 3 Modifies Lysine Residues to Facilitate Oligomerization of Mannan-Binding Lectin

**DOI:** 10.1371/journal.pone.0113498

**Published:** 2014-11-24

**Authors:** Maija Risteli, Heli Ruotsalainen, Ulrich Bergmann, Umakhanth Venkatraman Girija, Russell Wallis, Raili Myllylä

**Affiliations:** 1 Faculty of Biochemistry and Molecular Medicine, University of Oulu, Oulu, Finland; 2 Department of Diagnostics and Oral Medicine, Institute of Dentistry, University of Oulu, Oulu, Finland; 3 Medical Research Center Oulu, Oulu University Hospital and University of Oulu, Oulu, Finland; 4 Biocenter Oulu, University of Oulu, Oulu, Finland; 5 Biocenter Oulu, Mass Spectrometry Core Facility, University of Oulu, Oulu, Finland; 6 Department of Infection, Immunity, and Inflammation, University of Leicester, Leicester, United Kingdom; 7 Department of Biochemistry, University of Leicester, Leicester, United Kingdom; Boston University Goldman School of Dental Medicine, United States of America

## Abstract

Lysyl hydroxylase 3 (LH3) is a multifunctional protein with lysyl hydroxylase, galactosyltransferase and glucosyltransferase activities. The LH3 has been shown to modify the lysine residues both in collagens and also in some collagenous proteins. In this study we show for the first time that LH3 is essential for catalyzing formation of the glucosylgalactosylhydroxylysines of mannan-binding lectin (MBL), the first component of the lectin pathway of complement activation. Furthermore, loss of the terminal glucose units on the derivatized lysine residues in mouse embryonic fibroblasts lacking the LH3 protein leads to defective disulphide bonding and oligomerization of rat MBL-A, with a decrease in the proportion of the larger functional MBL oligomers. The oligomerization could be completely restored with the full length LH3 or the amino-terminal fragment of LH3 that possesses the glycosyltransferase activities. Our results confirm that LH3 is the only enzyme capable of glucosylating the galactosylhydroxylysine residues in proteins with a collagenous domain. In mice lacking the lysyl hydroxylase activity of LH3, but with untouched galactosyltransferase and glucosyltransferase activities, reduced circulating MBL-A levels were observed. Oligomerization was normal, however and residual lysyl hydroxylation was compensated in part by other lysyl hydroxylase isoenzymes. Our data suggest that LH3 is commonly involved in biosynthesis of collagenous proteins and the glucosylation of galactosylhydroxylysines residues by LH3 is crucial for the formation of the functional high-molecular weight MBL oligomers.

## Introduction

Lysyl hydroxylase 3 (LH3) is a multifunctional enzyme possessing three enzyme activities; lysyl hydroxylase (LH) (E.C. 1.14.11.4), collagen galactosyltransferase (GT) (E.C. 2.4.1.50) and glucosyltransferase (GGT) (E.C. 2.4.1.66) activities [1],[2]. Thereby, LH3 is able to catalyze the formation of glucosylgalactosylhydroxylysine (Glc-Gal-Hyl) residues, which are unique posttranslational modifications of collagens and collagenous proteins [3]. The other lysyl hydroxylase isoenzymes, LH1 and LH2, have only hydroxylation activities [3]. Recent studies show that LH3 is located not only in the ER, but also in the extracellular space, and that the GGT activity of serum originates from LH3 [4]. The presence of glycosyltransferase activities of LH3 in the extracellular space has been shown to be important for cell growth and viability [5]. Recently, mutations in the human LH3 gene (*PLOD3*) were shown to cause a severe connective tissue disorder with features that overlap with a number of collagen disorders. The patient lacked the urinary secreted glycosylated collagen cross-links and had dramatically reduced GGT activity in serum [6].

LH3 knockout studies in mice demonstrate that the loss of LH3 leads to embryonic lethality due to disruption in the formation of basement membranes [7],[8] and further analysis indicated that the absence of LH3 glycosyltransferase activities are responsible for the lethality [8]. The lack of LH3 leads to loss of all Glc-Gal-Hyl residues in collagens I, IV and VI and prevents the assembly and secretion of type IV and VI collagens [9]. In addition, the mutated LH activity, one out of three activities of LH3, leads to underglycosylation of collagen IV and VI, which is detected as abnormal distribution and aggregation of these collagens in mouse tissues [9]. Interestingly, even a moderate decrease in the amount of LH3 in heterozygous LH3 knockout mice and mouse embryonic fibroblasts (MEFs) causes abnormalities in the deposition and organization of extracellular matrix [10] suggesting that hydroxylation and glycosylation of collagens are important for their structural role in tissues.

In addition to 28 different collagen types [11], a short collagenous domain is also present in over 20 other proteins belonging to the C1q-domain containing (C1qDC) proteins, collectins, ficolins and the collagenous transmembrane protein families [12]–[14]. There is increasing evidence that lysine modifications also contribute to the structure and function of these other proteins. Mannan-binding lectin (MBL) is one of the collectin family proteins, which is a key component of the innate immune system by initiating the lectin pathway of complement activation [15],[16]. The collagenous domain of the MBL contains four lysine residues that are hydroxylated and further glycosylated to glucosylgalactosylhydroxylysines [17],[18]. MBL subunits form oligomers inside the producing cell, which are subsequently secreted in to the serum. The oligomers assemble through disulphide bonds at the N-terminal end of the subunits [15],[16], but indirect evidence suggests that the Glc-Gal-Hyl residues may mediate or modulate the oligomerization process which is essential for function [19]. The larger oligomers (trimers and tetramers of trimeric subunits) are most effective forms of MBL both with respect to glycan recognition of microbial surfaces and for complement activation [19]–[21]. Mutation of Glc-Gal-Hyl residues leads to reduction in MBL secretion, formation of large functional oligomers and activation of the complement pathway [20], however, the importance of the sugar residues towards these activities is not known.

We have previously shown the LH3 modifies the lysine residues in the collagenous domain of adiponectin, an insulin-sensitizing hormone, and thus affects the oligomerization and secretion of adiponectin [22]. In this study we have investigated whether LH3 is also responsible for the lysine modifications present in the collagenous domain of MBL. Our results indicate that LH3 is needed for the complete hydroxylysine glucosylation and thereby to efficient oligomerization of rat MBL-A to form the larger oligomers that are necessary for efficient pathogen recognition and complement activation.

## Materials and Methods

### Ethical statement

All mouse experiments were approved by the National Animal Ethics Committee of Finland and Laboratory animal center of Oulu university (License ESLH-2007-10387/Ym-23 and 036/10, respectively). Blood samples were collected by orbital bleeding under medetomin-ketamin terminal anesthesia.

### Mouse and cell lines

The generation of LH3 knockout and LH mutant mouse lines has been described previously [8]. The mice have been backcrossed to the C57BL/6 strain for 6 to 9 generations and were maintained under 12-hour light and dark cycles with ad libitum access to water and a standard chow diet. LH3^−/−^ knockout (KO) mouse embryonic fibroblasts (MEFs), LH mutant (LH) and wild type (WT) MEFs were derived as previously described [9]. All cell lines were cultured in Dulbecco's Modified Eagle Medium (DMEM) (Invitrogen) supplemented with 10% fetal calf serum (Promocell), penicillin/streptomycin, and 50 µg/ml ascorbic acid at 37°C in a humidified atmosphere of 95% air and 5% CO_2_.

### Production of recombinant mannan-binding lectin and LH3

Mammalian expression vectors pcDNA3 (Invitrogen) encoding a untagged or a carboxy-terminally FLAG-tagged rat MBL-A [23] and pcDNA3 containing the full length human LH3 or the amino-terminal part of LH3 to the amino acid 290 with a LH3 signal peptide and a c-Myc-tag at the amino-terminus [5],[24] were transfected into LH3^−/−^ knockout, LH mutant and wild type MEFs using a MEF 1 Nucleofector Kit and Amaxa Nucleofector technology (Lonza). The following day the culture medium was changed to serum free DMEM, penicillin/streptomycin, and 50 µg/ml ascorbic acid, and the cells were cultured for 48 h. The medium was collected and concentrated by Amicon Ultra centrifugal filters (10 kDa MWCO, Millipore).

### Immunoblot analysis

In order to analyze the amount or molecular weight of MBL-A, 0.5 µl of mouse serum, equal volumes of concentrated cell culture medium or 40 µg of soluble protein of mouse liver homogenate were loaded onto a 12 or 18% SDS-PAGE. Mouse liver was homogenized with 50 mM Tris-HCl pH 7.5, 150 mM NaCl, 1% Triton X-100, 1% Igepal and 0.1% SDS buffer including Complete EDTA-free protease inhibitor cocktail (Roche), and disrupted by brief sonication. The cell debris was removed by centrifugation. The protein concentrations were measured with Protein assay (Bio-Rad). The proteins were separated under reducing conditions by SDS-PAGE and transferred to an Immobilon-P transfer membrane (Millipore). For analysis of MBL-A oligomers, 5 µl of mouse serum or equal volumes of concentrated cell culture medium were separated under non-reducing and non-heat-denaturing conditions on a 10% SDS-PAGE and transferred as above. The membranes were blocked with 5% milk powder in Tris buffered saline −0.1% Tween 20 and incubated with rabbit polyclonal rat anti-MBL-A [20] or mouse anti-α-tubulin (Sigma) followed by a horseradish peroxidase-conjugated anti-rabbit IgG (P.A.R.I.S. Biotech) or anti-mouse IgG (P.A.R.I.S. Biotech), respectively. Immunocomplexes were visualized using an ECL, ECL+ or ECL prime detection system (GE Healthcare) and Molecular imager ChemiDoc XRS+ (Bio-Rad). Quantification of MBL-A levels was performed with Image Lab software (Bio-Rad).

### Mass Spectrometry analysis

The FLAG-tagged rat MBL-A was affinity purified using the anti-FLAG M2 affinity gel (Sigma) and eluted with the FLAG peptide (Sigma) or 2× SDS-PAGE loading buffer as described by the manufacturer. Proteins were fractionated on SDS-PAGE, and the band that corresponded to MBL-A on the Coomassie brilliant blue-stained gel was analyzed by MALDI Tof mass spectrometry as described earlier [22].

### Gel filtration chromatography

The different oligomers of MBL-A in 75 µl of mouse serum and in concentrated cell culture medium of three to four 58 cm^2^ culture dishes were fractionated by an Äkta purifier chromatography system (GE Healthcare) using a Superdex 200 10/300 GL gel filtration column (GE Healthcare) and eluted with 150 mM NaCl, 50 mM phosphate buffer, pH 7.2 at a flow rate of 0.5 ml/min. Fractions of 0.2 ml were collected, concentrated with speed-vac devise and the amount of MBL-A in the fractions was determined by immunoblot analysis under reducing and denaturing conditions as described above.

## Results

### Lysyl hydroxylase 3 is essential for the glucosyltion of MBL-A

**Figure 1:**
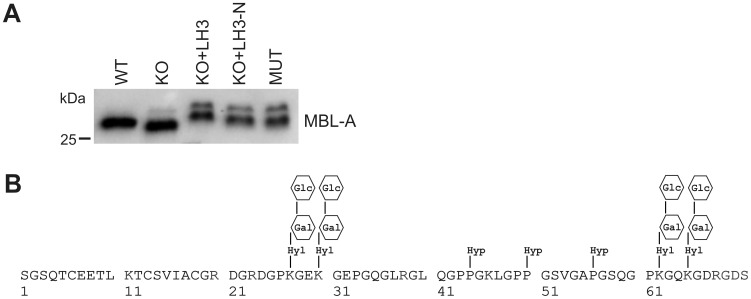
Immunoblot analyses of rat MBL-A produced in LH3 manipulated cell lines. (A) The immunoblot analysis of recombinant rat MBL-A monomers from the concentrated medium of wild type, LH3^−/−^ knockout and LH mutant MEF cells suggests differences in posttranslational modifications of MBL-A. Production of MBL-A with the full length LH3 or the amino-terminal fragment of LH3 restored the mobility shift on SDS-PAGE. The MBL-A produced with LH3 had a higher molecular weight than wild type protein, suggesting overglycosylation of lysines. Representative samples are shown. (B) Schematic picture of the collagenous domain of rat MBL-A shows the positions of posttranslational modifications of lysine and proline residues reported earlier. Abbreviations: WT = wild type; KO = LH3^−/−^ knockout; LH3 = full length LH3; LH3-N = amino-terminal fragment of LH3; MUT = LH mutant; Hyl = hydroxylysine; Gal = galactosyl; Glc = glucosyl; Hyp = hydroxyproline.

Our recent data show that LH3 catalyzes formation of Glc-Gal-Hyl residues in collagens [9] and in collagenous domain of adiponectin [22]. Similar lysine modifications are also present in MBL, the first component of the lectin pathway of complement activation [17],[18]. In order to determine how the manipulation of LH3 activities affects the lysine modifications of MBL-A, our LH3 MEF cell lines were transiently transfected with the construct encoding rat MBL-A. We used LH3^−/−^ knockout MEFs, where the LH3 protein and its three enzyme activities are totally absent, and LH mutant MEFs, where only one enzyme activity, the lysyl hydroxylase activity of LH3 is missing due to substitution Asp669Ala [8],[9]. In addition to rat MBL-A, LH3^−/−^ knockout MEFs were co-transfected with the construct of the full length human LH3 (LH3) or a 30 kDa amino-terminal fragment (LH3-N), which possess both glycosyltransferase activities of LH3 [5]. Immunoblot analysis showed that recombinant MBL-A produced in LH3^−/−^ knockout MEFs (Fig. 1A, KO) migrated faster than the protein produced in wild type MEFs (Fig. 1A, WT). When MBL-A was produced together with LH3 in LH3^−/−^ knockout MEFs (Fig. 1A, KO+LH3) the mobility shift disappeared and MBL-A seemed to have an even higher molecular weight than wild-type protein. Recombinant MBL-A produced in LH mutant (Fig. 1A, MUT) or in LH3^−/−^ knockout MEFs with the amino-terminal fragment of LH3 (Fig. 1A, KO+LH3-N) had a similar molecular weight compared with wild type. The immunoblot analysis indicated differences in the posttranslational lysine modifications of MBL-A produced in LH3^−/−^ knockout MEFs, which could be restored by recombinant LH3 or even its amino-terminal fragment possessing glycosyltransferase activities.

In order to further analyze the posttranslational modifications, FLAG-tagged rat MBL-A was affinity purified and tryptic peptide mixtures were analyzed by mass spectrometry. Recombinant rat MBL-A contains four lysine residues in the collagenous domain that are hydroxylated and further glycosylated (Fig. 1B) [19]. These residues fall within two tryptic peptides, which have theoretical molecular masses of 1524.776 (amino acids 24–38) and 2047.068 Da (amino acids 47–68) (Table 1). Both peptides can contain two Glc-Gal-Hyl residues, at position 27 and 30, and 62 and 65, resulting in the peptides of 2204.978 and 2727.269 Da respectively. In addition, the peptide with amino acids 47–68 has been shown to contain two hydroxyprolines [19] to give a fully modified peptide with the theoretical mass of 2759.259 Da. In our mass spectrometric analyses of recombinant rat MBL-A produced in wild type MEFs (Table 1, Fig. S1 and Table S1, WT) we detected a peptide with mass 2759 Da, in which lysines 62 and 65 were modified to Glc-Gal-Hyl and two hydroxyprolines were also present. In addition, comparison of the experimentally derived mass spectrum with the theoretical one revealed a peptide, which covers amino acids 47–68 with masses of 2727 and 2743 Da suggesting that prolines are not always fully hydroxylated and relative frequencies of these three peptides were similar. The peptide with mass of 2204 was not detected; instead a peptide covering amino acids 24–38 was present with a mass 1864 Da, which suggests that there is variation in modification of lysines 27 and 30. Variation was further supported by the presence of a peptide containing an internal uncleaved arginine spanning residues 21–38. This peptide was present with masses of 2193 and 2533 Da, which corresponds to the presence of one or two Glc-Gal-Hyl residues, respectively. Calculation of relative frequencies of these peptides revealed that in the most cases only one lysine of the two candidates is modified.

**Table 1:** Mass spectrometry identification of peptides and modifications from tryptic digests of recombinant rat MBL-A.

Position	Theoretical mass (Da) MH+	Observed mass (Da) MH+	Mass difference (ΔDa)	Modification	WT	KO	KO+LH3	KO+LH3-N	MUT
21–38	1852.926	1852.929	0	27, 30 not modified				+	
		1852.922	0						+
		1868.936	16.01	1× Hyl/Hyp				+	
		1868.910	15.984						+
		1884.897	31.971	27, 30 Hyl or 1× Hyl, 1× Hyp					+
		2193.041	340.115	1× Glc-Gal-Hyl	+				
		2193.035	340.109					+	
		2193.010	340.084						+
		2208.991	356.065	1× Glc-Gal-Hyl, 1× Hyl/Hyp			+		
		2209.016	356.090						+
		2225.008	372.082	2× Gal-Hyl, 1× Hyp		+			
		2533.121	680.195	27, 30 Glc-Gal-Hyl	+				
		2533.105	680.179				+		
		2533.137	680.211					+	
		2533.094	680.168						+
24–38	1524.776	1524.784	0	27, 30 not modified				+	
		1524.767	0						+
		1540.761	15.985	1× Hyl/Hyp					+
		1556.779	32.003	27, 30 Hyl or 1× Hyl, 1× Hyp		+			
		1556.754	31.978						+
		1572.777	48.001	27, 30 Hyl, 1× Hyp		+			
		1718.829	194.053	1× Gal-Hyl, 1× Hyl/Hyp		+			
		1734.818	210.042	1× Gal-Hyl, 1× Hyl, 1× Hyp		+			
		1864.876	340.100	1× Glc-Gal-Hyl	+				
		1864.885	340.109					+	
		1864.871	340.095						+
		1880.856	356.080	1× Glc-Gal-Hyl, 1× Hyl/Hyp			+		
		1880.860	356.084						+
		1896.881	372.105	27, 30 Gal-Hyl, 1× Hyp		+			
		2204.961	680.185	27, 30 Glc-Gal-Hyl			+		
28–38	1127.580	1127.594	0	27 not glycosylated, 30 not modified		+			
		1127.574	0					+	
		1127.569	0						+
		1143.578	15.998	27 not glycosylated, 30 Hyl or 1× Hyp		+			
		1143.556	15.976						+
		1159.587	32.007	27 not glycosylated, 30 Hyl, 1× Hyp		+			
		1321.638	194.058	27 not glycosylated, 30 Gal-Hyl, 1× Hyp		+			
47–62	1405.743	1405.754	0	62 not modified				+	
		1405.729	0						+
		1421.737	15.994	1× Hyl/Hyp				+	
		1421.725	15.982						+
		1437.754	32.011	62 Hyl, 1× Hyp or 2× Hyp		+			
		1437.743	32.000					+	
		1437.724	31.981						+
47–68	2047.068	2435.147	388.079	62, 65 Gal-Hyl, 2× Hyp		+			
		2419.139	372.071	1× Glc-Gal-Hyl, 1× Hyl, 1× Hyp					+
		2727.243	680.201	62, 65 Glc-Gal-Hyl	+				
		2727.269	680.201				+		
		2743.273	696.205	62, 65 Glc-Gal-Hyl, 1× Hyp	+				
		2743.248	696.180				+		
		2743.268	696.200					+	
		2759.261	712.193	62, 65 Glc-Gal-Hyl, 2× Hyp	+				
		2759.240	712.172				+		
		2759.275	712.207					+	
		2759.247	712.189						+

Abbreviations: WT = wild type; KO = LH3^−/−^ knockout; LH3 = full length LH3; LH3-N = amino-terminal fragment of LH3; MUT = LH mutant; Hyl = hydroxylysine; Gal = galactosyl; Glc = glucosyl; Hyp = hydroxyproline.

Glc-Gal-Hyl 340 Da, Gal-Hyl 178 Da, Hyl/Hyp 16 Da.

Analysis of MBL-A produced in LH3^−/−^ knockout MEFs (Table 1, Fig. S1 and Table S1, KO) revealed that the peptides found in wild type were undetectable. Instead, a new set of peptides with unique masses was detected. In peptides covering amino acids 24–38 and 47–68 mass difference between LH3^−/−^ knockout and wild type corresponded to the loss of Glc units in Glc-Gal-Hyl residues. Furthermore, peptides spanning amino acids 28–38 and 47–62 suggest that lysine 27 is not always galactosylated and there is variation in hydroxylation of lysines 30 and 62. Interestingly, we saw peptides covering amino acids 21–38, 24–38 and 28–38 with masses that corresponds to the presence of an additional hydroxyproline in position 33 and they were also the most frequent peptides covering amino acids 24–38. In human MBL, the proline corresponding to residue 33 of rat MBL-A is largely modified [18]; the masses observed suggest that in LH3^−/−^ knockout MEFs this proline residue is hydroxylated.

When MBL-A was produced together with the full length LH3 in LH3^−/−^ knockout MEFs (Table 1, Fig. S1 and Table S1, KO+LH3), we detected peptides with similar masses and relative frequencies as found in MBL-A produced in wild type cells, indicating that while other enzymes can at least partially compensate for the lysyl hydroxylase and galactosyltranseferase activities, LH3 is the only enzyme able to glucosylate the galactosylhydroxylysines. In this sample the peptide covering amino acids 24–38 was present with mass 2204, which shows that both lysines 27 and 30 are more frequently modified than in wild type, which also explains the higher molecular mass seen on immunoblots. Furthermore, even production of MBL-A with the amino-terminal fragment of LH3 (Table 1, Fig. S1 and Table S1, KO+LH3-N) was able to restore the presence of Glc units in Gal-Hyl residues, confirming that the amino-terminal fragment functions as glucosyltransferase *in cellulo* even though frequency of peptides with Glc-Gal-Hyl residue was lower compared with WT and KO+LH3 sample. In addition, in this sample multiple species were detected for each MBL-A peptide, revealing more variation in modification than with the full length LH3. MBL-A produced in LH mutant (lacking lysyl hydroxylase activity of LH3) MEFs (Table 1, Fig. S1 and Table S1, MUT) was trypsinized to several peptides, which showed that four lysine residues were modified to Glc-Gal-Hyl residues, but frequency of peptides with Glc-Gal-Hyl residue was much lower compared with WT and KO+LH3 sample. Overall, there was similar variation in lysine modification as seen in MBL-A produced in LH3^−/−^ knockout MEFs and in presence of amino-terminal fragment of LH3. Our mass analyses thus indicates that all three enzymatic activities of LH3 are needed in order to get precisely modified lysine residues in MBL-A. Whereas the lysyl hydroxylase and collagen galactosyltransferase activities can be partially compensated by other cellular enzymes, only LH3 can catalyze the glucosyltransferase activity within the collagenous domain of MBL. Our mass spectrometry analyses also explain the differences seen in the mobility of recombinant MBL-A proteins on immunoblot.

### Oligomerization of recombinant MBL-A is defective in LH3^−/−^ knockout MEFs

**Figure 2:**
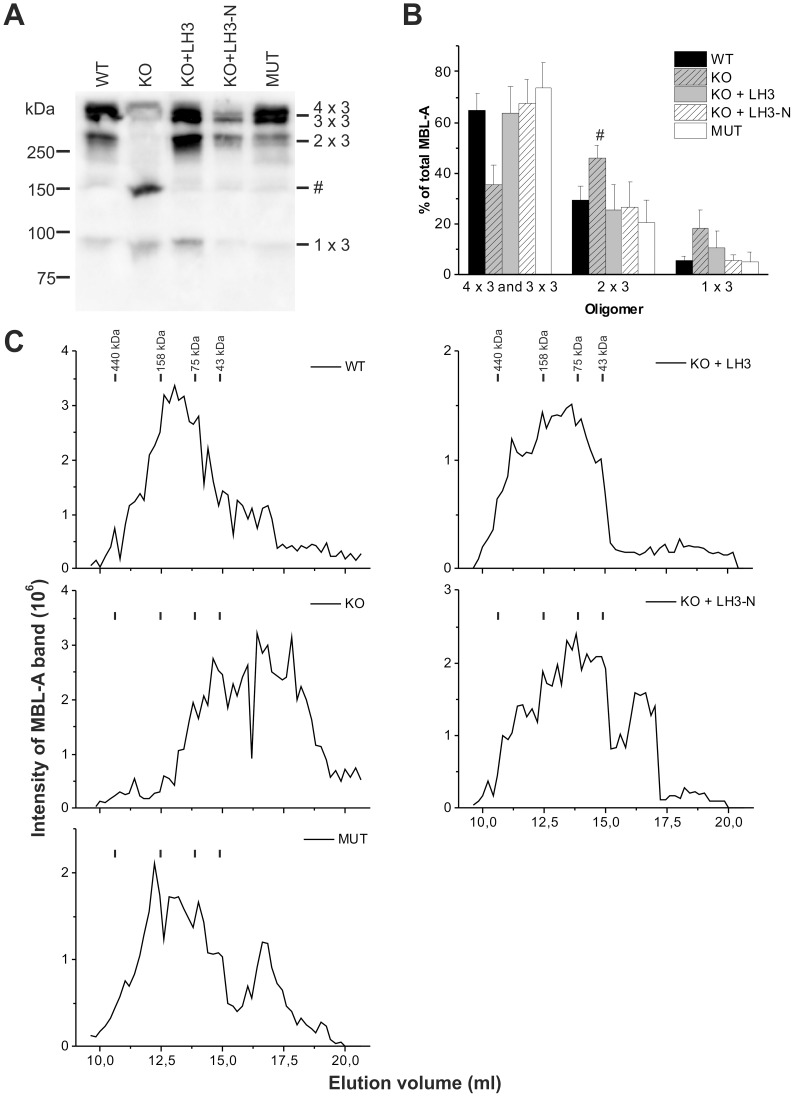
Oligomer distribution of recombinant MBL-A is altered in LH3^−/−^ knockout MEF cells. (A) Recombinant rat MBL-A produced in LH3 manipulated MEFs was separated under non-reducing and non-heat-denaturing conditions on SDS-PAGE and immunoblotted. Different oligomeric pattern was seen in MBL-A produced in LH3^−/−^ knockout MEFs compared with the other samples. The migration positions of covalent oligomeric forms of MBL-A are indicated on the right. Band marked with # represents four-chain covalent species of polypeptides. Representative samples are shown. (B) Quantification of MBL-A bands from immunoblots confirmed the change in the oligomer distribution of MBL-A produced in LH3^−/−^ knockout MEFs. Intensity of MBL-A band around molecular marker 150 kDa (marked with #) was quantified instead of the 2×3 band in LH3^−/−^ knockout MEFs. The values represent the average ± SD of six to eight experiments. (C) The oligomeric forms of recombinant rat MBL-A produced in LH3 manipulated MEFs were separated with a gel filtration chromatography and quantified from immunoblots. The gel filtration elution profile of MBL-A produced in LH3^−/−^ knockout MEFs also differed from wild type. Double transfection with MBL-A and LH3 constructs normalized the elution profile in LH3^−/−^ knockout MEFs. Equal volumes of concentrated cell culture media were used in all analysis. The average elution profiles of four to five experiments are shown. The elution positions of molecular weight markers are indicated. Abbreviations: WT = wild type; KO = LH3^−/−^ knockout; LH3 = full length LH3; LH3-N = amino-terminal fragment of LH3; MUT = LH mutant.

Normally in serum, MBL is found as oligomers of identical polypeptide chains. During biosynthesis, three chains assemble into trimeric subunits, which associate within the endoplasmic reticulum to form larger oligomers that are stabilized by interchain disulfide bonds. MBL is mainly found as dimers, trimers and tetramers of subunits in human and rodent serum, with humans also possessing small amounts of larger oligomers up to hexamers [15],[16]. The mutation of lysine residues 27 and 30 to arginine in rat MBL-A has been shown to reduce the formation of functional tetrameric and trimeric oligomers of MBL-A [20], but the reason for defective oligomerization is not known. In theory, the mutations themselves (i.e. loss of the lysine residues or insertion of the arginine side chain), the loss of hydroxylation of the lysine or loss of one or both sugar residues could disrupt oligomerization. In order to evaluate the role of glucosylation in oligomerization of MBL-A, samples were separated under non-reducing and non-heat-denaturing conditions on SDS-PAGE and immunoblotted. The major bands were quantified on immunoblots and the oligomer distribution of MBL-A was calculated. The tetrameric and trimeric oligomers migrated very closely each other and were quantified as one band. Rat MBL-A produced in wild type (Fig. 2A and B, WT) and LH mutant MEFs (Fig. 2A and B, MUT) was mostly detected as dimers, trimers and tetramers of subunits with similar distributions to each other. In LH3^−/−^ knockout MEFs, however, the oligomeric pattern of MBL-A differed greatly from the wild type (Fig. 2A and B, KO). The amounts of tetramers and trimers was ∼half that of normal and a new band was observed at ∼150 kDa (46% of the total MBL) corresponding to four covalently linked MBL-A polypeptides, revealing that the normal pattern of disulphide bonding is disrupted. When MBL-A was produced together with the full length LH3 (Fig. 2A and B, KO+LH3) or the amino-terminal fragment (Fig. 2A and B, KO+LH3-N) in LH3^−/−^ knockout MEFs, normal oligomerization was restored and the distribution of oligomers was similar to wild type.

Recombinant MBL-A proteins were also separated by gel filtration. Fractions were concentrated and the amount of MBL-A polypeptide in the fractions was determined by immunoblot analysis under reducing and denaturated conditions and quantified as described in Materials and Methods (Fig. 2C). The elution profiles of MBL-A produced in wild type (Fig. 2C, WT) and LH mutant MEFs (Fig. 2C, MUT) resembled each other. However, reduced oligomerization of MBL-A was observed in protein secreted from LH3^−/−^ knockout MEFs (Fig. 2C, KO), with the major peak eluting ∼3.5 ml later from the column and continuing ∼2.5 ml later than for the wild type. Synthesis of MBL-A in the presence of full length LH3 (Fig. 2C, KO+LH3) or its amino-terminal fragment (Fig. 2C, KO+LH3-N) in LH3^−/−^ knockout MEFs was able to restore the oligomerization pattern of MBL-A. Thus our data suggest that the glucose units of the Glc-Gal-Hyl residues are essential for efficient oligomerization of MBL-A.

### The amount of MBL-A is reduced in the serum of LH mutant mice

**Figure 3:**
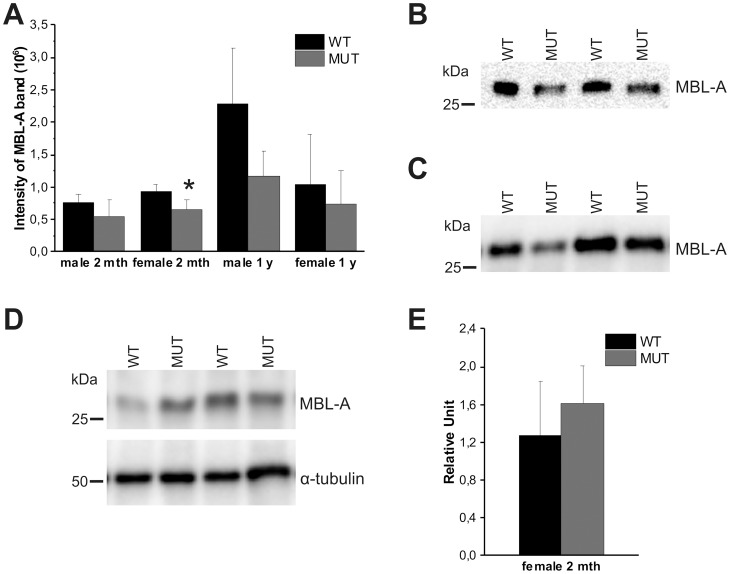
Amount of MBL-A in serum and liver of LH mutant mice. (A) Amount of MBL-A was reduced in the serum of LH mutant mice (MUT) compared with the corresponding wild type mice (WT). Amount of MBL-A was analyzed from the serum of the 2 months old male (n = 4 WT, 4 MUT) and female (n = 4 WT, 4 MUT) and 1 year old male (n = 4 WT, 3 MUT) and female (n = 5 WT, 6 MUT) by immunoblots. The values represent the average ± SD of the samples. MBL-A monomers in the serum of 2 months old female (B) and 1 year old male (C) wild type and LH mutant mice did not show significant differences in electrophoretic mobility. (D) The immunoblot analysis of liver homogenate (40 µg of soluble protein) of wild type and LH mutant male mice did not show differences in mobility of MBL-A monomers. (E) The amount of MBL-A in liver of LH mutant mice is higher than in wild type. Amount of MBL-A in liver was analyzed with immunoblots and α-tubulin was used as control to normalize the quantities of protein. In immunoblots B, C and D two representative wild type and LH samples are shown. MBL-A in LH mutant mice does not show doublet band, differing from the results of rat MBL-A overexpressed in cell culture system (Fig. 1A). The values in figure A and E represent the average ± SD of the samples. P values were calculated using unpaired homoscedastic student t-test with two-tailed distribution. * p<0.05.

We have shown recently that LH3 modifies the lysine residues in the collagenous domain of adiponectin and that circulating adiponectin levels were significantly reduced in LH mutant mice [22]. Due to the lethality of LH3^−/−^ knockout [8] we have used our LH mutant mice, in which the lysyl hydroxylase activity of LH3 is inactivated with a point mutation Asp669Ala, to study the effect of missing hydroxylysine residues catalyzed by LH3. In order to determine whether small differences in lysine modifications seen in our mass spectrometry analysis with recombinant proteins affect the level of MBL-A, we analyzed sera of our LH mutant mice (Fig. 3). The amount of MBL-A in serum of 2 month- and 1 year old LH mutant female and male mice was reduced to 51% to 73% of the levels of wild type mice (Fig. 3A) but with a statistical significance (p<0.05) observed only in 2 month old female mice. No obvious difference in the mobility of MBL-A from the mutant mice was seen when serum samples were compared with those from wild-type when separated on SDS-PAGE (Fig. 3B and C).

In previous work, mutation of Lys27 and Lys30 to Arg in the collagenous domain of rat MBL-A (and thereby the absence of modified lysine residues) lead to a reduction in the rate of secretion as MBL was retained for longer within producing cells [20]. In order to see whether the reduction in the amount of circulating MBL-A was due to reduced synthesis or secretion, the amount of MBL-A was measured in liver, where MBL-A is primarily synthesized [25]. The amount of MBL-A in liver of 2 months old female LH mutant mice was about 130% of wild type mice (Fig. 3E), suggesting that the production of MBL-A is normal or even slightly increased compared with the wild type mice. This suggests that the reduction of MBL-A seen in sera of 2 month old LH mutant female mice is most likely due to inefficient secretion from liver as a result of the reduced total level of lysine hydroxylation.

### MBL-A forms normally oligomeric structures in LH mutant mice

**Figure 4:**
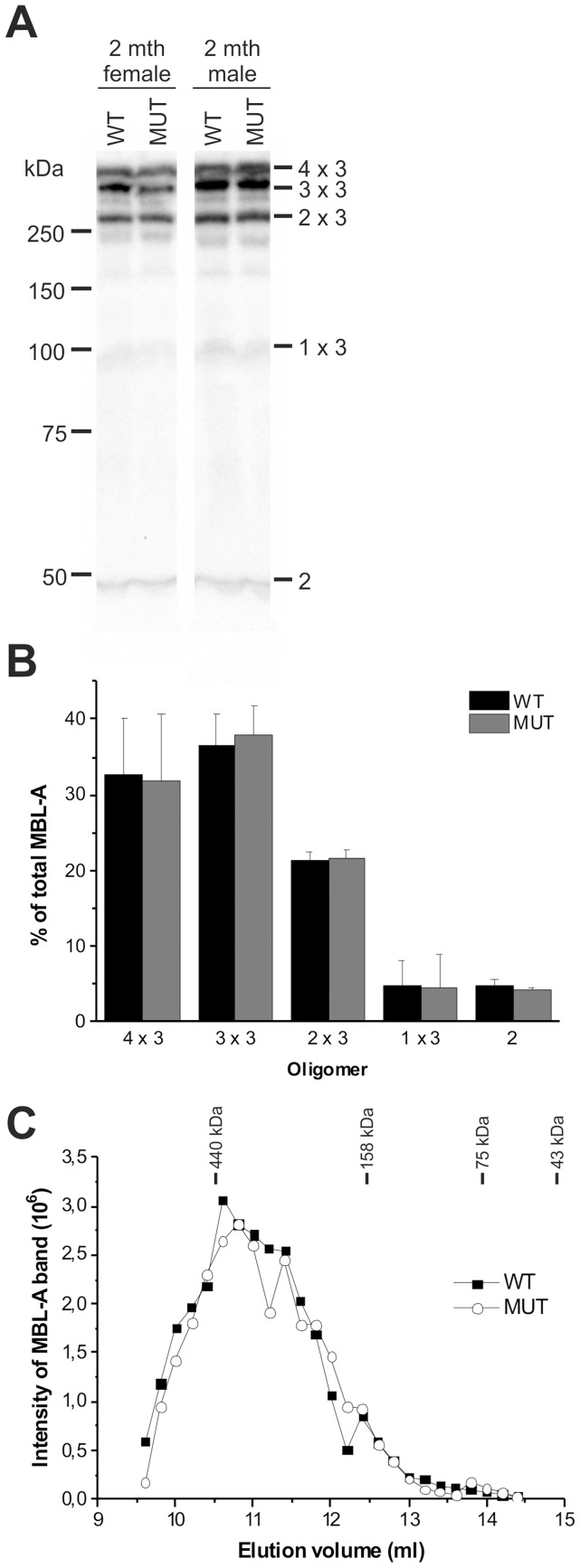
LH mutant mice form similar MBL-A oligomers as wild type mice. (A) In serum of 2 months old female and male LH mutant (MUT) and wild type (WT) mice, MBL-A was mainly seen as tetramers, trimers and dimers, when serum samples were separated under non-reducing and non-heat-denaturing conditions on SDS-PAGE and immunoblotted. Only small amount of monomers (1×3) was present in serum. The migration positions of covalent oligomeric forms of MBL-A are indicated on the right. Band marked with 2 represent two-chain covalent species of polypeptides. Representative samples are shown. (B) The distribution of the MBL-A oligomers was unchanged in serum of 2 months old female LH mutant mice compared with wild type (n = 4 WT, 4 MUT). Intensity of MBL-A oligomers was quantified from immunoblots and the level of oligomeric form was calculated as a proportion of total intensity of MBL-A. The values represent the average ± SD of the serum samples. (C) The elution profile of MBL-A in serum of 1 year old LH mutant mice was quite similar to the elution profile of wild type mice. The oligomeric forms were separated with gel filtration chromatography and quantified from immunoblots. The average elution profiles of three mice (WT and MUT) are shown. The elution positions of molecular weight markers are indicated.

In order to analyze the MBL-A oligomers assembled in LH mutant mice, the serum samples were separated under non-reducing and non-heat-denaturing conditions on SDS-PAGE and immunoblotted. As seen in Fig. 4A, in 2 month-old mouse serum, both wild type and mutant MBL-A assembled to dimers, trimers and tetramers (Fig. 4A), which resembled the oligomer pattern of recombinant rat MBL-A [20]. The five major bands on immunoblots were quantified in order to calculate the ratio of different oligomers. The results showed that the oligomer distribution was unchanged in LH mutant females (Fig. 4B) and males (data not shown) compared with the corresponding wild type. Furthermore, we analyzed MBL-A oligomers in the serum of 1 year old male mice by gel filtration (Fig. 4C). In the elution profiles, no difference in the amounts of oligomers was seen in LH mutant compared with the corresponding wild type. Our data suggests therefore that other LH isoforms can compensate the LH activity of LH3 and thus MBL-A can form normal oligomers in the mice lacking the LH activity of lysyl hydroxylase 3. This data thereby confirms the results with recombinant MBL-A that glucosyltransferase, not lysyl hydroxylase activity of LH3 is important in determining the oligomerization of MBL-A.

## Discussion

Our previous results have revealed that the glucosyltransferase activity of lysyl hydroxylase 3 (LH3) is especially important for the complete collagen lysine modifications [8],[9]. So far the glucosyltransferase activity of LH3 has not been discovered to be compensated by other enzymes. Recently, we also showed that absence of LH3 abolishes the glucosylation of galactosylhydroxylysine residues and disturbs oligomerization of adiponectin, a hormone containing a short collagenous domain with similar lysine modifications as found in collagens [22]. In order to evaluate whether LH3 is an enzyme commonly modifying lysine residues of proteins with a short collagenous domain we analyzed the posttranslational modifications and oligomerization status of MBL-A in our LH3 manipulated cell and mice lines.

Our results indicate that the recombinant rat MBL-A produced in LH3 knockout cells lacks glucosylation of the four lysines in the collagenous domain but these residues were hydroxylated and further galactosylated similar to the adiponectin produced in LH3 knockout cells [22]. Production of MBL-A in cells containing recombinant LH3 or the amino-terminal fragment (i.e. glycosyltransferase activities of LH3) restored the glucosylation, which indicate that LH3 is essential for glucosylation of galactosylhydroxylysine residues. This has also been reported by others: the suppression of LH3 decreased the glucosylation of collagen I [26],[27]. Our data also confirms that LH1 and LH2 can probably compensate lysyl hydroxylase activity of LH3 and hydroxylated lysines can be further galactosylated with other enzymes. It has been shown elsewhere, that collagen β(1-*O*)galactosyltransferases, GLT25D1 and GLT25D2 are able to transfer galactose to serum MBL [28]. In addition, GLT25D1, MBL and LH3 has been shown to have comparable sub-cellular localization after co-transfection [29], suggesting that GLT25D1 could galactosylate MBL-A in our LH3 knockout cells.

The work described here shows that efficient oligomerization of MBL is dependent on the glucosyltransferase activity of LH3. Oligomerization of serum proteins with a short collagenous domain is essential for their function. The defense collagens (MBL, C1q, collectins and ficolins) all form bouquet-like structures with multiple subunits to enable high avidity binding to microbial pathogens. The collagenous stems in C1q, MBL, collectin-11 and serum ficolins also provide the correct framework for binding to their associated proteases (C1r and C1s in the classical pathway and MASPs in the lectin pathway) and to trigger activation of complement on pathogen recognition [30],[31]. In this study MBLs, lacking terminal glucose units, fail to assemble correctly with altered disulphide bonding pattern and reduced oligomerization. The key factor controlling the oligomerization of MBL during biosynthesis is the formation of the interchain disulphide bonds within the cysteine-rich N-terminal region [32],[33]. The bulky glycosylated hydroxylysines are assumed to decrease the electrostatic and hydrophobic interactions between molecules and thereby decreasing non-specific intermolecular interactions [34]. According to our results it is possible that the glucosylated galactosylhydroxylysines of MBL are needed to create an optimal distance between subunits in order to form the disulphide bonds stabilizing the MBL oligomers. Alternatively, addition of the sugars may provide a target for scaffolding lectins within the endoplasmic reticulum to facilitate oligomerization. Sugar recognition is important in the secretory pathway of many glycoproteins. For example, the lectins and molecular chaperones calnexin and calreticulin promote folding, oligomeric assembly and quality control of glycoproteins within the endoplasmic reticulum [35].

MBL-A in the serum of LH mutant mice and the recombinant rat MBL-A produced in LH mutant cells were shown to have similar molecular weight as wild type suggesting normal modifications of lysine residues. However, our mass spectrometric analyses of recombinant MBL-A in LH mutants revealed more variation in the amount of lysine hydroxylation and glycosylation when compared with wild type, the posttranslational modifications thus not being precisely identical with the wild type molecule. Nevertheless, MBL-A in mouse serum and the recombinant rat MBL-A in LH mutant formed similar oligomeric structures with no obvious changes in distribution compared with wild type. This is in agreement with earlier results showing, that mutation of one of the lysine residues did not affect the formation of high-molecular weight MBL oligomers, while mutation of two residues totally abolished the formation of higher oligomers [20]. In addition, loss of lysyl hydroxylase activity of LH3 in our LH mutant mice leads to decreased amounts of MBL-A in serum, probably due to decreased secretion from producing cells. This finding is consistent with earlier results that showed that mutation of two hydroxylated and glycosylated lysine residues in collagenous domain of rat MBL-A lead to reduced secretion of MBL-A [20]. The current data indicate that it is the lack of lysine modification rather than the mutations themselves (Lys to Arg) that is responsible for the altered phenotype previously observed [20]. This result is also in agreement with our adiponectin data, which showed that secretion of adiponectin was decreased in LH mutant mice. The secretion of another adipokine leptin, which does not contain a collagenous domain or modified lysine residues, was unchanged in the blood circulation of LH mutant mice [22]. Thus LH3 is likely to be important for efficient secretion of other oligomeric collagenous proteins such as ficolins and C1q.

In conclusion, in this study we have shown for the first time that LH3 is essential for correct biosynthesis of MBL. Lack of the posttranslational modification of lysine residues, especially glucosylation of galactosylhydroxylysyl residues, reduces the formation of the tetrameric and trimeric oligomers of MBL-A, which are the most efficient forms for activating complement *in vivo*.

## Supporting Information

Figure S1 **MALDI Tof mass spectra of tryptic peptide mixtures.** The purified recombinant rat MBL-A produced in wild type, LH3^−/−^ knockout and LH mutant MEFs were in gel digested with trypsin and the peptide mixtures were analyzed with MALDI Tof mass spectrometry. Analysis of MBL-A produced in LH3^−/−^ knockout MEFs revealed a set of fragments with unique masses compared with wild type recombinant MBL-A. Production of MBL-A together with the full length LH3 or the amino-terminal fragment of LH3 in LH3^−/−^ knockout MEFs restored the set of peptides comparable with wild type. MBL-A produced in LH mutant MEFs was mainly trypsinized to fragments with similar masses as found in wild type. Abbreviations: WT = wild type; KO = LH3^−/−^ knockout; LH3 = full length LH3; LH3-N = amino-terminal fragment of LH3; MUT = LH mutant.
(TIF)

Table S1 **Relative frequency of peptides from the tryptic digest of recombinant rat MBL-A.**
(PDF)
