# Supplementary figures and images for: Lysyl Hydroxylase 3 Modifies Lysine Residues to Facilitate Oligomerization of Mannan-Binding Lectin
Source: PLoS One. 2014 Nov 24;9(11):e113498. doi: 10.1371/journal.pone.0113498 (PMC4242627; doi:10.1371/journal.pone.0113498)

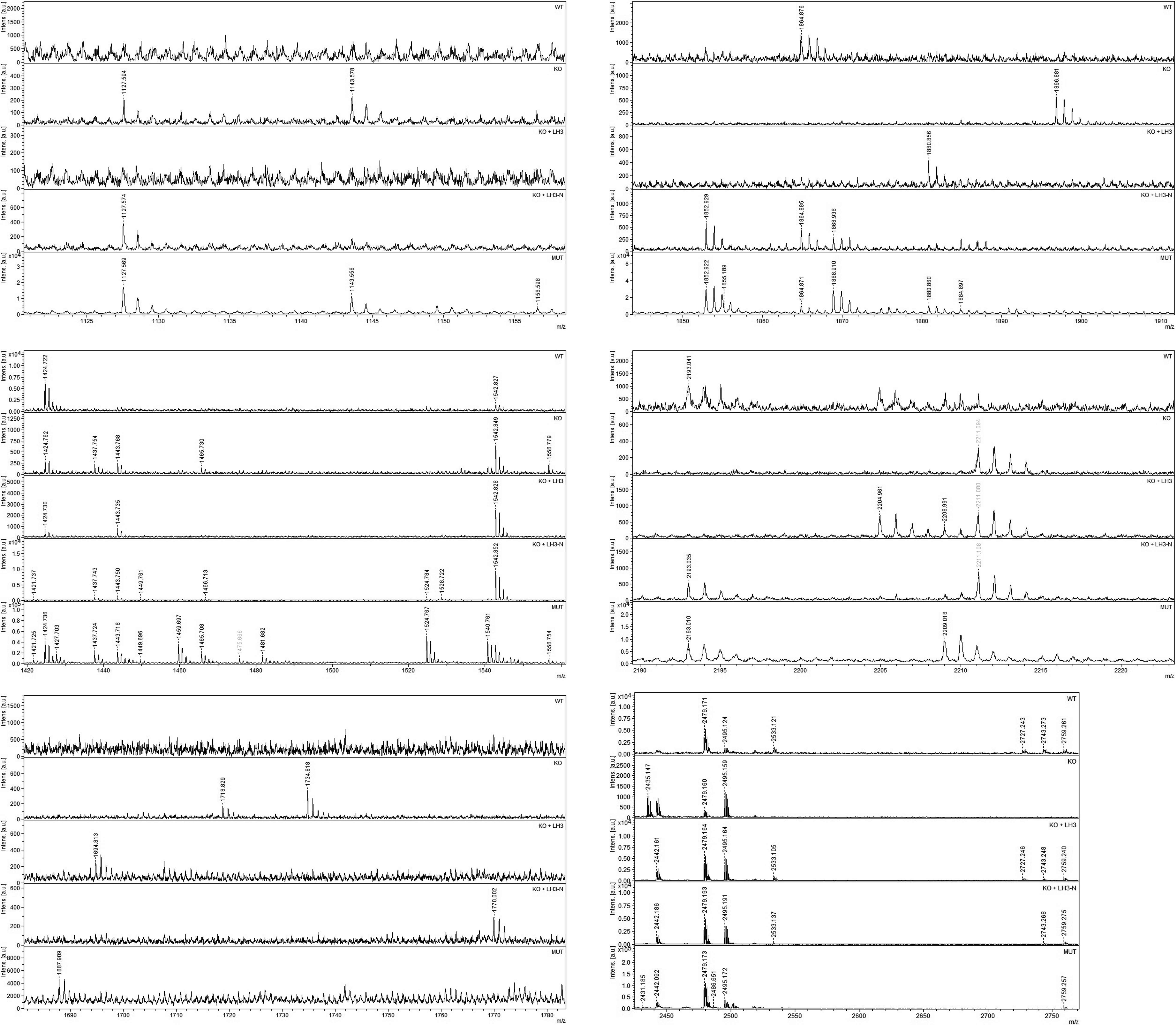

Supplement: Figure S1 — MALDI Tof mass spectra of tryptic peptide mixtures. The purified recombinant rat MBL-A produced in wild type, LH3−/− knockout and LH mutant MEFs were in gel digested with trypsin and the peptide mixtures were analyzed with MALDI Tof mass spectrometry. Analysis of MBL-A produced in LH3−/− knockout MEFs revealed a set of fragments with unique masses compared with wild type recombinant MBL-A. Production of MBL-A together with the full length LH3 or the amino-terminal fragment of LH3 in LH3−/− knockout MEFs restored the set of peptides comparable with wild type. MBL-A produced in LH mutant MEFs was mainly trypsinized to fragments with similar masses as found in wild type. Abbreviations: WT = wild type; KO = LH3−/− knockout; LH3 = full length LH3; LH3-N = amino-terminal fragment of LH3; MUT = LH mutant. (TIF) [file pone.0113498.s001.tif]
