# Supplementary material for: Lysyl Hydroxylase 3 Modifies Lysine Residues to Facilitate Oligomerization of Mannan-Binding Lectin
Source: PLoS One. 2014 Nov 24;9(11):e113498. doi: 10.1371/journal.pone.0113498 (PMC4242627; doi:10.1371/journal.pone.0113498)
Supplement: Table S1 — Relative frequency of peptides from the tryptic digest of recombinant rat MBL-A. (PDF) [file pone.0113498.s002.pdf]

Table SI Relative frequency of peptides from the tryptic digest of recombinant rat MBL-A

Position	Peptide	Theoretical mass (Da) MH+	Modification	Relative frequency of the peptide (%)#				
				WT	KO	KO + LH3	KO + LH3-N	MUT
21-38	21-38	1852.926	27, 30 not modified	nd	nd	nd	21	14
			1x Hyl/Hyp	nd	nd	nd	11	12
			27, 30 Hyl or 1 x Hyl, 1 x Hyp	nd	nd	nd	nd	2
			1 x Glc-Gal-Hyl	26	nd	nd	17	3
			1 x Glc-Gal-Hyl, 1x Hyl/Hyp	nd	nd	12	nd	7
			2x Gal-Hyl, 1 x Hyp	nd	6	nd	nd	nd
			27, 30 Glc-Gal-Hyl	31	nd	35	5	1
	24-38	1524.776	27, 30 not modified	nd	nd	nd	17	20
			1x Hyl/Hyp	nd	nd	nd	nd	15
			27, 30 Hyl or 1 x Hyl, 1 x Hyp	nd	6	nd	nd	3
			27, 30 Hyl, 1 x Hyp	nd	7	nd	nd	nd
			1x Gal-Hyl, 1 x Hyl/Hyp	nd	5	nd	nd	nd
			1x Gal-Hyl, 1 x Hyl, 1 x Hyp	nd	13	nd	nd	nd
			1 x Glc-Gal-Hyl	42	nd	nd	17	3
			1 x Glc-Gal-Hyl, 1x Hyl/Hyp	nd	nd	18	nd	6
			27, 30 Gal-Hyl, 1 x Hyp	nd	24	nd	nd	nd
			27, 30 Glc-Gal-Hyl	nd	nd	34	nd	nd
	28-38	1127.580	27 not glycosylated, 30 not modified	nd	8	nd	12	9
			27 not glycosylated, 30 Hyl or 1x Hyp	nd	9	nd	nd	4
			27 not glycosylated, 30 Hyl, 1x Hyp	nd	14	nd	nd	nd
			27 not glycosylated, 30 Gal-Hyl, 1 x Hyp	nd	9	nd	nd	nd

(Continued on next page)

Table SI (continued)

Position	Peptide	Theoretical mass (Da) MH+	Modification	Relative frequency of the peptide (%)# WT	KO	KO + LH3	KO + LH3-N	MUT
47-68	47-62	1405.743	62 not modified	nd	nd	nd	12	20
			1x Hyl/Hyp	nd	nd	nd	19	15
			62 Hyl, 1 x Hyp or 2 x Hyp	nd	19	nd	53	38
	47-68	2047.068	62, 65 Gal-Hyl, 2 x Hyp	nd	81	nd	nd	nd
			1 x Glc-Gal-Hyl, 1 x Hyl, 1 x Hyp	nd	nd	nd	nd	18
			62, 65 Glc-Gal-Hyl	29	nd	33	nd	nd
			62, 65 Glc-Gal-Hyl, 1 x Hyp	37	nd	29	4	nd
			62, 65 Glc-Gal-Hyl, 2 x Hyp	35	nd	37	12	9

Relative frequency of the peptide was calculated as a percentage of intensity from the total intensity of peptides on position 21-38 or 47-68.

Abbreviations: nd = not detected; WT = wild type; KO = $LH3^{-/-}$ knockout; LH3 = full length LH3; LH3-N = amino-terminal fragment of LH3; MUT = LH mutant; Hyl = hydroxylysine; Gal = galactosyl; Glc = glucosyl; Hyp = hydroxyproline.
